# Differential hippocampal shapes in posterior cortical atrophy patients: A comparison with control and typical AD subjects

**DOI:** 10.1002/hbm.22999

**Published:** 2015-10-13

**Authors:** Emily N. Manning, Kate E. Macdonald, Kelvin K. Leung, Jonathan Young, Tracey Pepple, Manja Lehmann, Maria A. Zuluaga, M. Jorge Cardoso, Jonathan M. Schott, Sebastien Ourselin, Sebastian Crutch, Nick C. Fox, Josephine Barnes

**Affiliations:** ^1^ Dementia Research Centre, Institute of Neurology, University College London London United Kingdom; ^2^ Centre for Medical Image Computing, University College London London United Kingdom

**Keywords:** hippocampus, shape, PCA, Alzheimer's disease, Alzheimer, classifier, support vector machine, atrophy, morphometry

## Abstract

Posterior cortical atrophy (PCA) is a neurodegenerative syndrome characterized by predominant visual deficits and parieto‐occipital atrophy, and is typically associated with Alzheimer's disease (AD) pathology. In AD, assessment of hippocampal atrophy is widely used in diagnosis, research, and clinical trials; its utility in PCA remains unclear. Given the posterior emphasis of PCA, we hypothesized that hippocampal shape measures may give additional group differentiation information compared with whole‐hippocampal volume assessments. We investigated hippocampal volume and shape in subjects with PCA (*n* = 47), typical AD (*n* = 29), and controls (*n* = 48). Hippocampi were outlined on MRI scans and their 3D meshes were generated. We compared hippocampal volume and shape between disease groups. Mean adjusted hippocampal volumes were ∼8% smaller in PCA subjects (*P* < 0.001) and **∼**22% smaller in tAD subject (*P* < 0.001) compared with controls. Significant inward deformations in the superior hippocampal tail were observed in PCA compared with controls even after adjustment for hippocampal volume. Inward deformations in large areas of the hippocampus were seen in tAD subjects compared with controls and PCA subjects, but only localized shape differences remained after adjusting for hippocampal volume. The shape differences observed, even allowing for volume differences, suggest that PCA and tAD are each associated with different patterns of hippocampal tissue loss that may contribute to the differential range and extent of episodic memory dysfunction in the two groups. *Hum Brain Mapp 36:5123–5136, 2015*. © **2015 The Authors Human Brain Mapping Published by Wiley Periodicals, Inc.**

## INTRODUCTION

Posterior cortical atrophy (PCA) is a clinicoradiological syndrome characterized by impairment of visuoperceptual, visuospatial, and other posterior cognitive functions and atrophy of the occipital, parietal, and occipito‐temporal cortices [Benson et al., 1988; Crutch et al., 2012]. PCA is most commonly caused by Alzheimer's disease (AD) [Alladi et al., 2007; Renner et al., 2004; Tang‐Wai et al., 2004] and is probably the most common atypical clinical presentation of AD [Dubois et al., 2014]. Although AD is the most common underlying pathology in PCA (>80%) [Alladi et al., 2007; Renner et al., 2004; Tang‐Wai et al., 2004] a number of cases have other underlying pathologies such as dementia with Lewy bodies (DLB), corticobasal degeneration, prion disease, and subcortical gliosis [Crutch et al., 2012]. Unlike typical AD (tAD), where memory loss is one of the earliest and most prominent symptoms, episodic memory function in PCA is initially relatively well preserved [Mendez et al., 2002; Tang‐Wai et al., 2004].

The hippocampus, known to play an important role in the formation of long‐term, consciously accessible memories [Mayes et al., 2007; Squire et al., 2007], is one of the earliest structures to atrophy in tAD. Recently, hippocampal atrophy, visually or volumetrically assessed using magnetic resonance imaging, has been included in diagnostic criteria for AD [Dubois et al., 2007; Hyman et al., 2012]. In addition, lower hippocampal volumes have been proposed as an enrichment strategy to select individuals at risk of developing clinical AD for trials of putative treatments [Hill et al., 2014]. Further, change in hippocampal volume is widely used as a biomarker in AD clinical trials [Fox et al., 2005].

Previous whole‐brain cross‐sectional MRI studies (which used voxel‐based morphometry to compare gray matter volume differences and FreeSurfer to compare cortical thickness) have shown distinct atrophy patterns in subjects with PCA as compared to tAD with PCA showing greater atrophy in the right occipital cortex [Whitwell et al., 2007] and in the posterior parietal cortex (most markedly in the right posterior parietal cortex) [Lehmann et al., 2011] compared with tAD subjects and tAD subjects showing greater atrophy in the left medial temporal lobe compared to PCA [Lehmann et al., 2011; Whitwell et al., 2007]. A reduction in gray matter volume was observed in the right hippocampus in PCA as compared with controls using voxel‐based morphometry [Whitwell et al., 2007]. However there has been no study to date that has specifically assessed hippocampal volume and/or shape differences in PCA subjects as compared with controls and tAD subjects. Evaluating the extent to which the hippocampus is affected in PCA may contribute to efforts to improve our understanding of the factors driving phenotypic heterogeneity in AD. More practically, the value of biomarkers such as hippocampal atrophy may also differ in PCA compared with typical AD, and have a bearing upon the question of whether to include individuals with PCA in clinical trials in which study outcome measures have been selected for patients with more typical amnestic or global clinical presentations [Crutch et al., 2012].

In this study, we aimed to: investigate hippocampal volume differences between PCA, tAD, and healthy controls; localize areas of hippocampal tissue loss; and investigate whether shape metrics give any additional group separation information above volume alone.

## METHODS

### Subjects

One hundred twenty‐four subjects were identified retrospectively from a clinical database at the Dementia Research Centre (PCA (*n* = 47), tAD (*n* = 29), and control subjects (*n* = 48)). All subjects have been described in a previous study of gray matter volume and cortical thickness in PCA and tAD [Lehmann et al., 2011]. Subjects required at least one T1 weighted volumetric MRI scan to be included in the study. All PCA patients fulfilled the clinical criteria for PCA proposed by Mendez et al. [2002] and Tang‐Wai et al. [2004] and more recently by Dubois et al., [2014] criteria for atypical AD, including evidence of posterior cortical dysfunction on neuropsychological assessment and atrophy on MRI. In addition, subjects were only included in the PCA group if there was no indication of another underlying pathology (such as DLB). Although the neuropsychological tests completed were not identical across all individuals, all PCA patients showed evidence of deficits (scored <5th percentile) in at least two tasks sensitive to parietal dysfunction—object perception (VOSP Object Decision test [Warrington and James, 1991a, 1991b]), spelling (Graded Difficulty Spelling test [Baxter and Warrington, 1994]), space perception (VOSP Number Location test [Warrington and James, 1991b]), and calculation (Graded Difficulty Arithmetic test [Jackson and Warrington, 1986])—and had relatively preserved episodic memory (>5th percentile on verbal and or visual Recognition Memory Tests [Warrington, 1984, 1996]). Those included in the tAD group fulfilled revised NINCDS‐ADRDA criteria for probable AD [Dubois et al., 2007; McKhann et al., 1984] and had significant episodic memory impairments (namely gradual and progressive change in memory function, objective evidence of significantly impaired episodic memory, and presence of medial temporal lobe atrophy) with episodic memory impairments quantified as performance <5th percentile on the verbal and visual Recognition Memory Tests [Warrington, 1984, 1996]).

### Image Acquisition

T1 weighted volumetric MR scans were acquired for all subjects on 1.5 T Signa scanners (General Electric, Milwaukee). All scans used an inversion recovery sequence and all but 7 of the scans consisted of 124 contiguous 1.5 mm coronal slices through the head. The remaining 7 consisted of 120 contiguous 1.5 mm coronal slices (5 PCA subjects and 2 tAD subjects). Since this was a retrospective cohort, there was some variation in the scan parameters and in‐plane resolutions of the MRI scans; See Table 1 for a breakdown of the imaging parameters by diagnostic group. The majority of subjects in each diagnostic group had an in‐plane resolution of 0.9 mm × 0.9 mm (including all 7 subjects with 120 coronal slices).

**Table 1:** MRI scan parameters by diagnostic group

MRI parameters		Controls^a^ (*n* = 48)	PCA (*n* = 47)	tAD (*n* = 29)
FOV (mm)		240–280	200–280	200–280
TR (ms)		11.7–15	11.7–15	13.6–15
TE (ms)		4.2–5.4	4.2–6.4	4.2–5.4
TI (ms)		650	650	650
Flip angle (degree)		13–20	13–20	15–20
% Phase FOV		75–100	75–100	75–100
Slice thickness (mm)		1.5	1.5	1.5
No. of subjects by in‐plane resolution (mm)	0.9 × 0.9	35	31	21
	1.1 × 1.1	4	1	3
	0.8 × 1.0	7	14	5
	0.8 × 1.3	0	1	0

a Scan parameters not available for 2 subjects.

### Image Processing

#### Brain, hippocampal, and total‐intracranial volume extraction

In‐house segmentations software [Freeborough et al., 1997] was used to segment whole‐brains and hippocampi.

Whole‐brain regions were segmented in native space using a semi‐automated technique [Freeborough et al., 1997] and were manually edited where necessary. These whole brain regions were used to generate a volume, and also to use in the subsequent registration step.

The MRI scans were then aligned to MNI space and resampled to produce isotropic voxels of 1 mm × 1 mm × 1 mm. The left and right hippocampi were manually segmented by experienced image analysts. The hippocampi were manually delineated using every coronal slice referencing a standard neuroanatomical atlas [Duvernoy, 2005] using a protocol that was largely similar to the EADC‐ADNI Harmonized Hippocampal Protocol (HarP) [Boccardi et al., 2015]. Our protocol includes the head, body, and full extent of the hippocampal tail. Two key differences between our protocol and HarP were (1) we excluded the white matter that separates the lateral ventricles from the gray matter of the hippocampus at the level of the hippocampal tail and (2) we excluded vertical digitations from the hippocampal head. In addition, we used a minimum threshold of 70% of the mean whole brain intensity (using the whole brain region transformed into MNI space) to determine the boundary between the CSF and hippocampus for improved consistency.

Estimated total intracranial volume (eTIV) was measured using Freesurfer [Buckner et al., 2004].

### Hippocampal Shape Analysis

The hippocampal regions generated by manual segmentation were used to analyze differences in shape between the subject groups. In this study, we used spherical harmonic (SPHARM) decomposition to represent hippocampal shape. Arbitrarily shaped but simply connected objects can be decomposed into a weighted series of SPHARM basis functions. SPHARM shape decompositions have the advantage of encapsulating both global and local shape features compactly. The SPHARM‐PDM (Spherical Harmonics‐Point Distribution Model) toolbox was used to calculate the coefficients of the SPHARM basis functions of the hippocampi [Styner et al., 2006]. For a more detailed description of the SPHARM processing steps used in this study see Appendix A.

### Statistics: Demographics

We compared age (at the time of the scan) between the diagnostic groups using linear regression analysis with age as the dependent variable and diagnostic group (PCA, tAD, or controls) as the independent variable. Fishers exact test was used to compare the gender distributions between the groups. An unpaired *t*‐test was used to compare Mini‐Mental State Examination (MMSE) scores between tAD and PCA subjects. All analyses were performed in Stata 12.0.

### Statistics: Brain and Hippocampal Volume Analyses

We compared whole brain and hippocampal volumes between the diagnostic groups using linear regression analyses. Brain or hippocampal volume was the dependent variable, diagnostic group (PCA, tAD or controls) was the independent variable, and we adjusted for mean‐centered age, gender, and mean‐centered head size. All analyses were performed in Stata 12.0.

### Statistics: Hippocampal Shape Analysis

We used the SurfStat toolbox for Matlab to perform statistical comparisons on the hippocampal shapes [Worsley et al., 2009]. We performed two analyses on the hippocampal shapes. In the first analysis, the distance between the surface of individual meshes and the mean mesh was the dependent variable, group was the independent variable and we adjusted for mean‐centered age, gender, and mean‐centered head size. This was in order to visualize whether there were any shape or volume differences in PCA subjects as compared to controls and tAD. The second analysis was like the first analysis, but we adjusted for mean‐centered hippocampal volume, instead of mean‐centered head size. This was in order to visualize shape differences that were not due to volume differences. We ran additional analyses adjusting for mean‐centered MMSE score and mean‐centered disease duration as well as mean‐centered age, gender, and mean‐centered head size for the PCA vs. tAD comparisons in order to determine whether the differences observed in these comparisons were independent of these measures of disease severity. All comparisons were corrected for multiple comparisons (family‐wise error FWE correction). Maps showing where there were significant differences in hippocampal surface morphology were generated along with effect size maps.

### Classification of Subjects Using Hippocampal Shape Features

To quantify the extent to which hippocampal shape differences described group differences, we used soft‐margin support vector machines (SVMs) [Cortes and Vapnik, 1995]. The python package sci‐kit learn was used for this purpose [Pedregosa et al., 2011]. Since we were interested in whether SPHARM coefficients were better able to distinguish groups than hippocampal volume alone we used two SVMs for each group‐wise comparison, one with the SPHARM coefficients as features and the other using just the left and right hippocampal volumes as features. Each subject had a total of 1,014 SPHARM coefficients (from both the left and right hippocampi) since we used decomposition up to degree 12 (see Appendix A). A nested cross‐validation approach was taken and is described in detail in Appendix B.

## RESULTS

### Participant Demographics

A total of 124 subjects were included in this study. See Table 2 for a summary of participant demographics. The mean age of tAD subjects was higher by approximately 5 years than in controls and PCA subjects (*P* < 0.02 in both comparisons). The mean MMSE scores was lower in tAD subjects than PCA subjects (*P* = 0.01). There was no difference in disease duration or gender distributions between the diagnostic groups (*P* > 0.4, both tests).

**Table 2:** Participant demographics

	Controls (*n* = 48)	PCA (*n* = 47)	Typical AD (*n* = 29)	*P*‐value
Age (years)	63.6 (9.7)	63.0 (7.0)	68.3 (8.4)	0.02^a^
% Male	31%	40%	45%	0.5^b^
MMSE score/30	N/A	21.2 (4.6)	18.3 (4.5)	0.01^c^
Disease duration (years)	N/A	4.9 (2.7)	5.3 (3.1)	0.6^c^

a Regression analysis.

b Fisher's Exact Test.

c Unpaired *t*‐test.

Mean (SD) unless otherwise stated.

### Brain and Hippocampal Volume Analysis

Both PCA subjects and tAD subjects had significantly smaller mean adjusted brain volumes than the controls (*P* < 0.001) but there was no significant difference in mean adjusted brain volume between the PCA subjects and the tADs (*P* = 0.3). PCA subjects were found to have significantly smaller mean adjusted hippocampal volumes on both the right and left sides as compared to controls (*P* ≤ 0.002, both comparisons) (see Table 3). They were however significantly larger than those seen in tAD subjects (*P* ≤ 0.001, both comparisons).

**Table 3:** Brain and hippocampal volumes (adjusted for age, gender and head‐size)

	Controls (*n* = 48)	PCA (*n* = 47)	Typical AD (*n* = 29)
Mean adjusted brain volume (cm^3^)	1131.7 [1111.5, 1151.9]	1005.8^a^ [9851.0, 1026.5]	1021.9^a^ [9957.5, 1048.1]
Mean adjusted left hippocampal volume (cm^3^)	3.2 [3.1, 3.3]	3.0^a^ [2.8, 3.1]	2.5^a,b^ [2.3, 2.6]
Mean adjusted right hippocampal volume (cm^3^)	3.3 [3.2, 3.4]	3.0^a^ [2.9, 3.2]	2.6^a,b^ [2.4, 2.7]

a *P* ≤ 0.001 as compared with controls.

*P* = 0.000 as compared with PCA.

Mean [Confidence Interval]

### Hippocampal Shape Analysis

#### Comparison of PCA and controls

**Figure 1:**
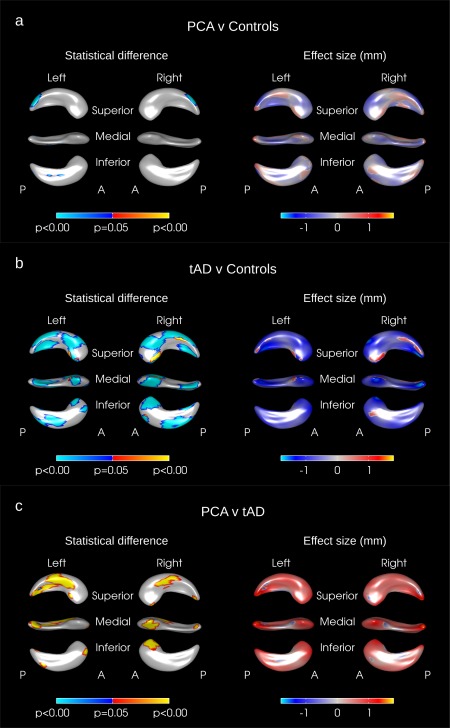
Hippocampal shape difference after adjusting for age, gender, and TIV in (**a**) PCA vs. controls, (**b**) tAD vs. Controls, (**c**) PCA vs. tAD. The color scale for statistical difference represents the FWE‐error corrected *P*‐values at a threshold of *P* = 0.05. Blue indicates areas where there was an inward deformation in (a) PCA as compared to tAD, (b) tAD as compared to controls, (c) PCA as compared to tAD whereas red/yellow indicates areas where there was an outward deformation. A = anterior, P = Posterior. [Color figure can be viewed in the online issue, which is available at http://wileyonlinelibrary.com.]

**Figure 2:**
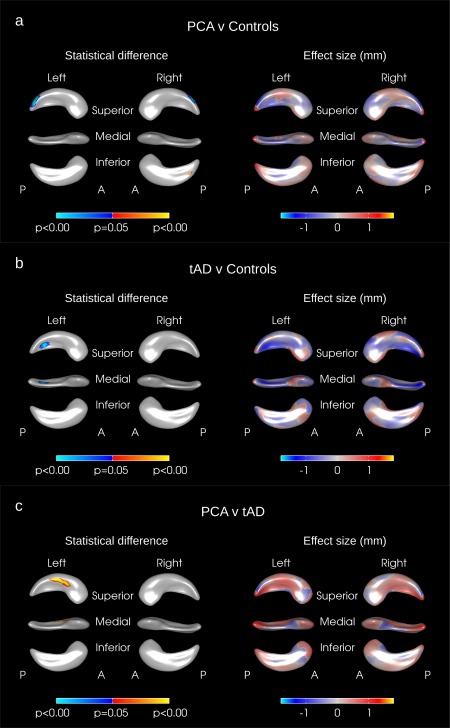
Hippocampal shape difference after adjusting for age, gender, and hippocampal volume in (**a**) PCA vs. controls, (**b**) tAD vs. Controls, (**c**) PCA vs. tAD. The color scale for statistical difference represents the FWE‐error corrected *P*‐values at a threshold of *P* = 0.05. Blue indicates areas where there was an inward deformation in (a) PCA as compared to tAD, (b) tAD as compared to controls, (c) PCA as compared to tAD whereas red/yellow indicates areas where there was an outward deformation. A = anterior, P = Posterior. [Color figure can be viewed in the online issue, which is available at http://wileyonlinelibrary.com.]

Significant differences in surface morphology were seen between PCA subjects and controls when adjusting for age, gender, and head size: these were largely confined to the posterior hippocampus with inward deformations in the hippocampal tail region on both the right and left sides of the PCA subjects compared to controls (see the blue regions in Fig. 1a). Significant inward deformations remained in the hippocampal tail regions of PCA subjects when adjusting for hippocampal volume rather than head size effectively identifying areas of focal loss or deformation over and above the global hippocampal volume loss (see blue regions in Fig. 2a).

#### Comparison of PCA and tAD

**Figure 3:**
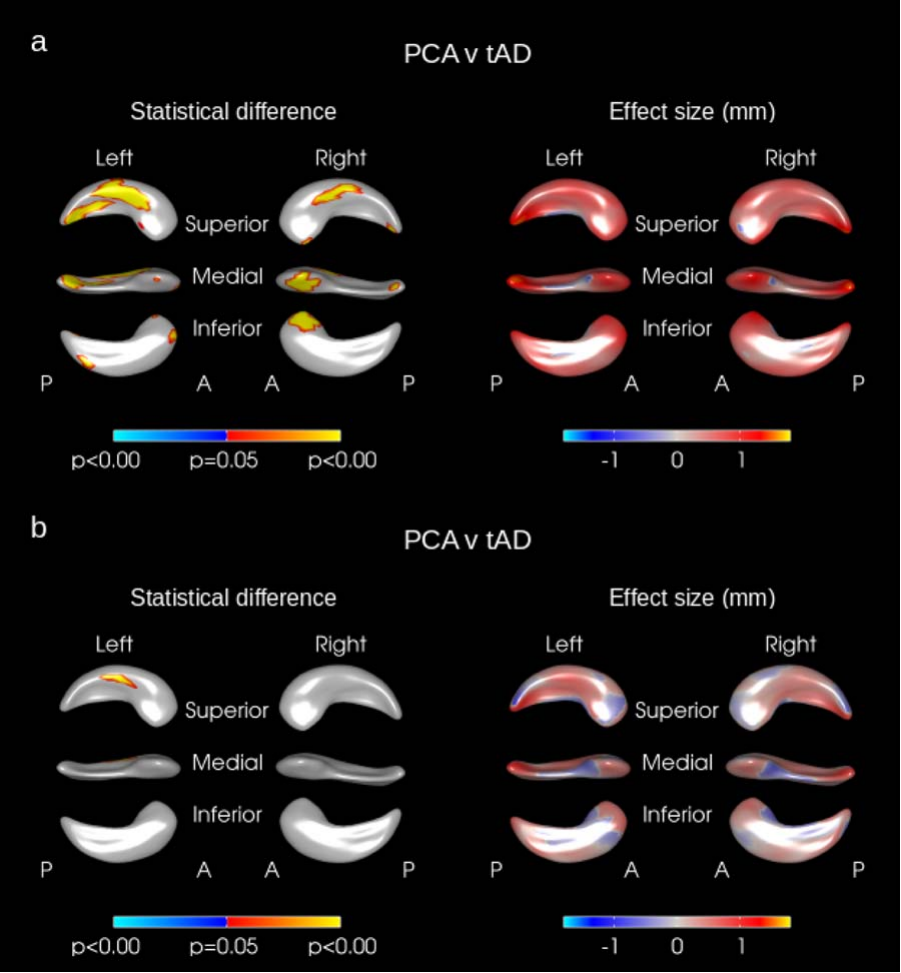
Hippocampal shape difference in PCA vs. tAD after adjusting for (a) age, gender, MMSE score, disease duration, and head size and (**b**) age, gender, MMSE score, disease duration, and hippocampal volume. The color scale for statistical difference represents the FWE‐error corrected *P*‐values at a threshold of *P* = 0.05. Blue indicates areas where there was an inward deformation in PCA as compared to tAD whereas red/yellow indicates areas where there was an outward deformation. A = anterior, P = Posterior. [Color figure can be viewed in the online issue, which is available at http://wileyonlinelibrary.com.]

Outward deformations in the mean right and left hippocampal surfaces of PCA subjects were seen compared with tAD in large areas across the whole of the hippocampus (see yellow/red regions in Fig. 1c). There were only very small regions where the mean surface of the tAD subjects had a significant outward deformation compared to PCA (see blue regions in Fig. 1c). When adjusting for hippocampal volume rather than TIV no significant differences remained on the right side but significant differences in hippocampal shape were still seen in the left hippocampus in the left superior body with PCA outwardly deformed compared to tAD (see Fig. 2c). The hippocampal shape differences observed between PCA and tAD subjects appear to be independent of disease severity as adjusting for MMSE score and disease duration made very little difference to the deformation patterns observed (see Fig. 3).

#### Comparison of tAD and Controls

In tAD, large areas of the mean left and right hippocampal surfaces were inwardly deformed as compared to controls when adjusting for age, gender, and head size (see Fig. 1b). The mean tAD hippocampal surface was inwardly deformed with respect to controls in most areas (see the blue regions in Fig. 1b) with some small regions where there was an outward deformation of the mean surface in tAD compared to controls (see the red/yellow regions in Fig. 1b). When adjusted for hippocampal volume however, only a small region of significant difference survived in the superior medial left hippocampal tail (see Fig. 2b) and there were no significant differences on the right side.

### Disease Classification Using SPHARM Coefficients

The accuracies, sensitivities, specificities, mean AUCs, *f*‐scores for each of the SVMs are shown in Table 4. In the PCA–control comparison, by using SPHARM coefficients only we were able to achieve a classification accuracy of 77% compared to 56% when using hippocampal volume information alone. McNemar's test showed that the SVM classifier using SPHARM coefficients significantly outperformed the classifier using hippocampal volumes (*P* = 0.002). In the controls–tAD and PCA–tAD comparisons, hippocampal volume alone was able to classify subjects as accurately as the SPHARM coefficients.

**Table 4:** Comparison of performance of SVM classifier using SPHARM coefficients vs SVM classifier using left and right hippocampal volumes as features

	SVM features	Sensitivity	Specificity	Accuracy	Mean AUC	*f*‐score	*P*‐value (McNemar's test)
PCA vs. Controls	SPHARM coefficients only	0.74	0.80	0.77	0.84	0.76	0.002
	Hippocampal volumes only	0.59	0.55	0.56	0.57	0.56	
PCA vs. tAD	SPHARM coefficients only	0.68	0.82	0.77	0.82	0.69	0.819
	Hippocampal volumes only	0.67	0.81	0.76	0.83	0.67	
Controls vs. tAD	SPHARM coefficients only	0.77	0.91	0.86	0.93	0.80	0.763
	Hippocampal volumes only	0.77	0.94	0.87	0.91	0.81	

*P*‐values are from McNemar's test.

## DISCUSSION

PCA subjects had significantly reduced (8% lower) mean adjusted hippocampal volumes (adjusted for age, gender and head size) compared to controls; it is interesting to note that the PCA subjects had relatively preserved episodic memory function despite this volume loss. The shape analyses pointed to the differences in surface morphology in PCA being relatively localized posteriorly with inward deformations seen in the hippocampal tail regions in comparison with controls.

The loss of hippocampal volume in PCA was much lower than that seen in typical amnestic AD (25% smaller hippocampi than controls). PCA subjects had significantly larger hippocampal volumes than tAD subjects and that was reflected in the shape differences reported. When adjusting for age, gender, and head size, large areas of outward deformations, likely representing regions of relatively preserved hippocampal tissue, were found in PCA subjects compared to tAD; these were mostly seen in the superior hippocampal body with some more minor differences in the tail and the head portion of the subiculum.

When we adjusted for hippocampal volume rather than head size, we still saw some significant shape differences in PCA subjects as compared to tAD (PCA>tAD) over a small area in the left hippocampus. Given that the majority of difference was removed by adjusting for hippocampal volume it is unsurprising that SPHARM coefficients did not aid in the classification of PCA subjects from tAD.

To our knowledge, this is the first study to report shape differences in the hippocampi of PCA subjects. These results suggest that although the hippocampi in PCA subjects are relatively preserved as compared to tAD, there is some tissue loss occurring in the hippocampi of PCA subjects compared with controls. The tissue loss appears to be most significant in the superior lateral hippocampal tail region, fitting with the posterior pattern (or gradient) of atrophy seen in these subjects. In addition, when adjusting for hippocampal volume, significant differences in surface morphology were still seen in PCA subjects. Consistent with this, the hippocampal SPHARM coefficients were better able to classify PCA subjects from controls than volume alone. Taken together these data indicate that there is a distortion of the shape of the hippocampi in PCA, which could be due to focal atrophy in the hippocampus as well as the tissue to which it is connected. Although the exact functional organization of the hippocampus remains unclear, it has been suggested that the posterior hippocampus supports detailed, context‐rich spatial [Hirshhorn et al., 2012] and autobiographical [Addis et al., 2004] memories, whilst the anterior hippocampus supports more “gist”‐like memories [Strange et al., 2014]). To date there has been no detailed characterization of memory function in PCA, but the present findings of a posterior–anterior gradient of hippocampal volume loss and shape change may predict qualitative as well as quantitative distinctions between memory processes in PCA and tAD.

As expected, we found significantly reduced hippocampal volumes in the tAD subjects as compared to controls: widespread significant inward deformations were seen across large areas of both the right and left hippocampi in tAD. Although it is difficult to precisely locate these inward deformations with respect to hippocampal subfields, in tAD these seem to approximate to the CA1 subfield as well as the anterior and posterior subiculum. A number of previous studies have compared hippocampal shapes in tAD and controls [Gerardin et al., 2009; Li et al., 2007; Lindberg et al., 2012; Shen et al., 2012; Thompson et al., 2004]. Our findings are in keeping with two previous studies that found inward deformations in tAD subjects across large areas of the both the left and right hippocampi [Gerardin et al., 2009; Shen et al., 2012]. One study found large areas of inward deformations on the left hippocampus, particularly in the hippocampal head as well as the superior tail region but found no differences in the right hippocampus [Li et al., 2007]; another study reported localized inwards deformations in the hippocampal head in tAD subjects, particularly on the left side [Thompson et al., 2004] whilst another study found some inward deformations in the body of the left hippocampus and a small area of inward deformation on the medial part of the right hippocampal head [Lindberg et al., 2012]. Differences in the numbers of subjects, disease severities, shape analysis methods, and hippocampal segmentation methods used may account for some of the different findings in these studies. When adjusting for hippocampal volume, we found no significant differences in shape on the right hippocampus and only a small region in the superior medial portion of the hippocampal tail on the left hippocampus. One other study [Shen et al., 2012] also investigated shape differences where the effect of volume was removed and, as in our study, found significant shape differences in the posterior hippocampus. The fact that most of the differences in shape were removed when adjusting for hippocampal volume suggests that in tAD there was generalized, diffuse tissue loss across the whole of the hippocampus. Indeed, we found that in this comparison, the SPHARM coefficients did not aid in the classification of tAD subjects from controls.

The fact that shape metrics helped separate PCA patients from controls suggests that they may be useful in addition to volume and could be explored in other diseases where diagnosis is difficult and subtle differences in atrophy patterns exist. In this study, shape metrics were no better than hippocampal volumes at distinguishing tAD subjects from controls. However, it is possible that the hippocampus does not atrophy uniformly during the tAD disease course. Indeed, previous studies have shown that the CA1 subfield is disproportionately affected in early AD [Chételat et al., 2008; Csernansky et al., 2005; La Joie et al., 2013; Mueller et al., 2010; Pluta et al., 2012; Wang et al., 2006] and that hippocampal subfields or hippocampal shape may be more sensitive at distinguishing MCI or very mild AD subjects from controls than whole‐hippocampal volume [Csernansky et al., 2005; La Joie et al., 2013; Mueller et al., 2010; Pluta et al., 2012]. Therefore, SPHARM coefficients may prove to be more useful at distinguishing controls from tAD at an earlier disease stage.

This study has a number of strengths. First, the hippocampi were segmented manually, including the full extent of the structure from tail to head. Secondly, although PCA is an atypical variant of AD, we had a reasonable number of cases to include in our analyses. The mean MMSE score was lower in the tAD subjects than in the PCA subjects, this reflects the weighting of the questions toward memory and orientation and the relative lack of questions relating the visual deficits experienced by PCA subjects. Brain volumes in the PCA and tAD subjects were not significantly different however suggesting similar levels of overall brain atrophy between the groups. Future studies with more detailed neuropsychological testing are required to investigate the inevitably complex relationship between clinical phenotype, cognition, and hippocampal shape and volume.

There were several limitations to this study that warrant discussion. First, the SPHARM‐PDM pipeline requires that the shapes being analyzed have spherical topology. In the case of one of the hippocampi from one of the subjects with tAD, the SPHARM‐PDM processing failed, perhaps because this hippocampus did not have spherical topology (this subject was therefore excluded from all analyses and from the demographics table). It could be that the failure rate is higher when comparing subjects with particularly pronounced atrophy or by use of automated techniques where borders of the hippocampal masks may not adhere to the spherical topological description. Secondly, we applied some smoothing to the segmented regions before the spherical parameterization. Therefore, it may be that some of the differences that do in fact exist are not found using this method since they have been attenuated. Thirdly, pathological confirmation of AD was only available in five of the PCA subjects and it may be that some of the remaining PCA subjects actually have a different underlying disease [Crutch et al., 2012]. Fourthly, the type of registration is an important consideration in interpreting the results regarding localization of tissue loss in any comparison. Other registration methods may align hippocampi differently and therefore localize deformations in other areas. Fifthly, the MRI scans used in this study were from a retrospective cohort with some variety in the scan parameters and in‐plane resolutions; ideally, all subjects would have identical imaging parameters. Although we do not believe that this would materially affect the results presented here, we cannot exclude this as a possibility and further studies using consistent imaging parameters would be required to confirm our findings. Sixthly, the images used were of limited resolution compared with the high‐resolution temporal lobe imaging which is achievable [Winterburn et al., 2013]. Given that the hippocampi are relatively small structures it may be that using higher resolution scans would enable the detection of more subtle shape differences between groups. Finally, caution is required when interpreting the results from shape analysis studies—a recent study indicated that the SPHARM‐PDM method of shape analysis might overestimate regions of significant difference [Gao et al., 2014]. We used stringent statistical methods (family‐wise error correction) in order to minimize false detection of differences where there were in fact none.

In conclusion, the hippocampal region is affected in PCA at a relatively earlier stage of the disease when memory is relatively preserved and produces posterior shape changes. We found reduced hippocampal volumes in PCA subjects as compared to controls—intermediate between controls and tAD. Whereas the macroscopic differences between tAD and control subjects were governed by volume rather than shape, as were the differences between PCA and tAD, most of the differences between PCA and controls are governed by shape differences (PCA smaller in the tail). This was further evidenced by shape (SPHARM) coefficients that were better able to distinguish healthy controls from PCA subjects than hippocampal volume alone suggesting that shape metrics are important descriptors of hippocampal differences in PCA as compared with controls.

## Figures and Tables

**Figure A1:**
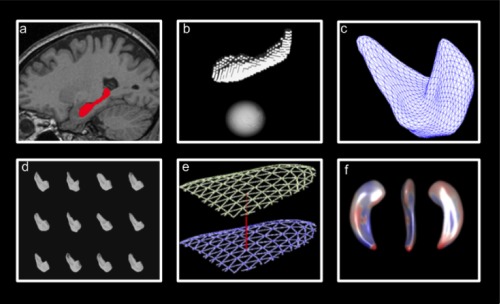
Summary of shape analysis procedure: (**a**) Hippocampi are segmented, holes filled and minimal smoothing applied, (**b**) raw surface meshes generated and spherical parameterizations are computed, (**c**) triangulated surface meshes are computed from spherical parameterizations, (**d**) surface meshes aligned (translation and rotation), (**e**) distance between mean mesh and individual meshes at each vertice is calculated, (**f**) statistical comparisons performed. [Color figure can be viewed in the online issue, which is available at http://wileyonlinelibrary.com.]

**Figure B1:**
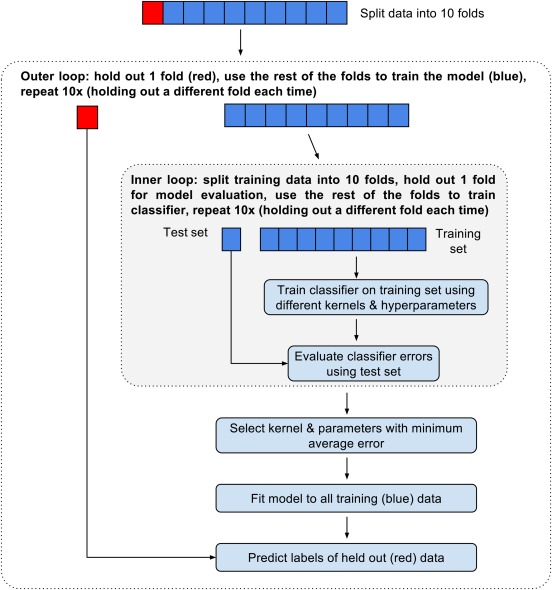
Nested 10‐fold cross‐validation procedure used for model tuning and evaluation. [Color figure can be viewed in the online issue, which is available at http://wileyonlinelibrary.com.]

## APPENDIX A SPHARM PROCESSING STEPS

Any structure with spherical topology can be represented by a weighted sum of spherical harmonic (SPHARM) functions. For a perfect representation of the original shape an infinite number of SPHARM basis functions would be required. In practice, the number of SPHARM basis functions used to represent the original shape is determined by a user‐defined parameter “Lmax,” the maximum degree of the SPHARM expansion. The greater Lmax is, more basis functions will be used in the representation and the finer the surface representation becomes. We chose to set Lmax = 12 as we felt that this provided a sufficient amount of detail.

The processing steps were as follows:

1. The hippocampi, that had been manually segmented in MNI space at an isotropic resolution of 1 mm, were binarized and resampled to an isotropic resolution of 0.5 mm. Interior holes were filled and a minimal smoothing operation was applied to ensure spherical topology (Fig. A1a). As described by Styner et al. [2006], the smoothing was a two‐step process: first a binary closing operation was applied followed by anti‐aliasing smoothing. The anti‐aliasing smoothing operation used in the SPHARM‐PDM package (ITK filter itk::AntiAliasBinaryImageFilter) smooths out jagged boundaries but uses the original binary surface as a constraint ensuring minimal loss in detail or structure (the smoothed surface is guaranteed to be within ± 3 voxels of the original surface) [Whitaker, 2000].
2. These pre‐processed binary segmentations were then transformed into raw surface meshes and spherical parameterizations for each of the hippocampal meshes were computed (Fig. A1b).
3. From the raw surface meshes and their spherical parameterizations, SPHARM (spherical harmonic) descriptions were computed and corresponding triangulated surface meshes were generated (Fig. A1c). These were all visually checked against the original manual segmentations to ensure that the segmentations were well represented by their SPHARM decompositions.
4. The triangulated surface meshes were then aligned to one (randomly selected) individual's hippocampal mesh using Procrustes alignment (translation and rotation only) and the triangulated surface meshes were then regenerated such that the vertices corresponded between each individual's mesh and the chosen reference mesh of the single subject (Fig. A1d). This was done for left and right sides separately.
5. A mean mesh was calculated from the aligned meshes (including the individual reference mesh used for alignment in the previous step) and the meshes were then aligned to the mean mesh using Procrustes alignment and again the individual meshes were regenerated such that the vertices corresponded between this mean mesh and each subject's meshes. Again, this was done for the left and right sides separately.
6. The meshes were loaded up side by side and the alignment was visually checked for alignment failures.
7. Finally, the triangulated surfaces meshes were converted from VTK format to MNI object format and imported into Matlab.

## APPENDIX B CLASSIFICATION OF SUBJECTS USING HIPPOCAMPAL SHAPE FEATURES

A nested cross‐validation approach was taken whereby the sample was split into 10 mutually exclusive stratified sets of approximately equal size. Figure B1 summarizes the nested cross‐validation process used. The classifier was trained and evaluated 10 times, once with each of the folds (data splits) as the test set and the remaining data used for training. In order to determine the best kernel (linear or radial basis function (RBF)) and kernel parameters, we used grid search and stratified 10‐fold cross‐validation (experimental evidence suggests 10‐fold cross‐validation is the best method for model selection [Kohavi, 1995 ]) on the training data each time. This sample splitting for kernel parameter choice was performed in order to avoid over‐fitting the data. Once the kernel and hyperparameters were tuned, the resulting SVM was fitted to the training data. Finally, the SVM was used to predict the labels of the test set on which the SVM was not trained. This process was repeated for each of the 10 folds. We used the same folds for the SVM with SPHARM coefficients as features and for the SVM with hippocampal volumes as features in order to be able to make comparisons. The accuracy (proportion of correctly classified subjects), sensitivity (proportion of true positives), and specificity (proportion of true negatives) were then calculated. We also chose to report the area under the ROC curve (AUC) and *f*‐score statistics since they both have advantages over accuracy when assessing the performance of a classifier. The AUC takes into account the decision value of the classifier which accuracy ignores. The *f*‐score is appropriate for imbalanced classes (where one class is under‐represented compared to another) and so is a better measure of the performance of a classifier than accuracy. Accuracy, sensitivity, and specificity are the same whether computing across all folds or whether taking the average of each of the folds. The AUC and *f*‐measure are different however when computing across all folds or when averaging over the folds. Previous work suggests that taking the mean AUC of each of the cross‐validation folds and computing the *f*‐score across all folds (as opposed to averaging) are less biased [Forman and Scholz, 2010]. Therefore, mean AUC and *f*‐score over all folds are reported in this study. Finally, in order to determine whether the SVM using the SPHARM coefficients was significantly better or worse at classification than the SVM using hippocampal volumes as features, we used the McNemar test [McNemar, 1947] since it has been shown to have a low type‐1 error [Dietterich, 1998].
